# Age‐specific over‐the‐top techniques for physeal sparing anterior cruciate ligament (ACL) reconstruction in skeletally immature patients: Current concepts for prepubescents to older adolescents

**DOI:** 10.1002/ksa.12607

**Published:** 2025-02-04

**Authors:** Alberto Grassi, Kyle A. Borque, Mitzi S. Laughlin, Matthew A. Tao, Stefano Zaffagnini

**Affiliations:** ^1^ II Clinica Ortopedica e Traumatologica, IRCCS Istituto Ortopedico Rizzoli Bologna Italy; ^2^ Department of Orthopedic Surgery Houston Methodist Hospital Houston Texas USA; ^3^ Houston Methodist Academic Institute Houston Texas USA; ^4^ Department of Orthopaedic Surgery and Rehabilitation University of Nebraska Medical Center Omaha Nebraska USA

**Keywords:** anterior cruciate ligament reconstruction, open physes, over‐the‐top ACL technique, paediatric

## Abstract

Tailored surgical strategies for anterior cruciate ligament (ACL) reconstruction in skeletally immature patients are presented, emphasizing techniques to minimize growth plate damage and ensure stability. As ACL injuries in youth increase, delaying surgery can lead to joint damage and poor recovery outcomes. Using magnetic resonance imaging‐based assessments of skeletal maturity, the authors propose the ‘over‐the‐top (OTT)’ approach with lateral tenodesis, adapted for three growth stages: prepubescents, young adolescents and older adolescents. For prepubescents, the extra‐physeal approach avoids growth plate drilling; for young adolescents, the supra‐physeal technique places tunnels above the growth plate; and for older adolescents, the trans‐physeal method mirrors adult techniques, as growth plates are closing. The minimally invasive OTT technique preserves hamstring insertion, ensures isometric graft placement, and allows for combined intra‐ and extra‐articular procedures to improve rotational control and protect the graft. Clinical outcomes highlight high return‐to‐sport rates, minimal growth disturbances, and low failure rates, although older adolescents show higher graft failures due to activity levels. Various adaptations of the OTT and lateral tenodesis techniques utilizing hamstring tendons provide promising solutions for addressing ACL injuries in skeletally immature patients, ranging from prepubescence to late adolescence. Assessing skeletal age and estimating remaining bone growth are essential for selecting the most appropriate surgical method. The biomechanical principles and positive clinical results observed across different patient groups highlight these techniques as effective, safe and attractive options for managing these challenging cases.

**Level of Evidence:**

Level IV.

**Abbreviations**
ACL: anterior cruciate ligament
ITB: iliotibial band
OTT: over‐the‐top

## INTRODUCTION

Anterior cruciate ligament (ACL) injuries in skeletally immature individuals, which pose a significant challenge for treating surgeons, are increasing [3, 42]. The desire to restore stability to the knee must be balanced against the potential risk of iatrogenic injury to the physes. Physeal injury can lead to coronal or sagittal plane deformity as well as shortening or lengthening of the affected limb [16, 28]. This risk rises as the amount of remaining growth increases. Older guidelines recommended treating skeletally immature patients with activity avoidance and postponing ACL reconstruction surgery until skeletal maturity [5]. While this is the only method to guarantee avoidance of damaging the physes, multiple studies have now shown that delayed surgical treatment of ACL injuries in young patients comes at the cost of increased meniscal and chondral damage, as well as lower return to sport [2, 8, 21, 34]. In addition, concomitant pathologies such as a bucket handle meniscus tear, full‐thickness chondral injury with a loose body or collateral ligament injuries requiring surgical treatment would be an indication for acute surgery, necessitating a safe and reproducible approach to address the ACL tear in these young patients.

Multiple surgical approaches have been developed in an attempt to restore stability while decreasing the risk of injury to the physes in skeletally immature patients [1, 27, 32]. These techniques can be divided into transphyseal and physeal‐sparing. Transphyseal techniques, which may be appropriate for patients near skeletal maturity, utilize soft tissue grafts with a more vertically oriented femoral tunnel to minimize damage to the femoral physis surface area. The drawbacks include violation of the physes, and the non‐anatomic vertical orientation of the ACL graft may lead to persistent rotational instability. Physeal‐sparing techniques can be either all‐epiphyseal or extra‐physeal [38]. All‐epiphyseal techniques utilize tunnels drilled in the tibial and femoral epiphyses, to avoid crossing the growth plates. While this approach has been shown to produce good clinical results, it is technically challenging and has a higher risk of damage to one of the physes. Finally, the extra‐physeal approach was popularized by Kocher and Micheli [18] who described using a strip of the ITB, left attached at Gerdy's tubercle, bringing it through the knee joint and fixing it to the anterior tibia. This creates an ACL reconstruction plus an anterolateral reconstruction to protect the intra‐articular graft.

It was with this in mind that the authors proposed modifications of a long‐used [45, 46] ‘over‐the‐top (OTT)’ and lateral tenodesis ACL reconstruction technique described by Marcacci et al. [24]. In addition to excellent results with more than 25 years of follow‐up, this technique also provides the benefit of being minimally invasive with decreased morbidity, preserved vascularization of the hamstring tendons as well as reproducible, isometric placement of the ACL graft. Evaluation of the patient's bone age, and thus the amount of growth remaining, is critical to determining which surgical approach is appropriate for each patient.

While there is consensus that the surgical technique should be tailored to the patient's skeletal age, there remains significant debate regarding how to estimate skeletal bone age as well as the ideal technique depending on how much growth is remaining [30]. This paper aims to provide a framework for the management of skeletally immature patients with ACL injury based on the authors' experience, developing an algorithm for the treatment and suggesting the most appropriate surgical technique for ACL reconstruction according to the skeletal age. The techniques described are modifications of Marcacci's OTT and lateral tenodesis ACL reconstruction technique [24].

## SKELETAL GROWTH AND BONE AGE ASSESSMENT

Understanding the fundamentals of overall skeletal and knee growth [17] is crucial when managing skeletally immature patients with ACL ruptures. When ACL reconstruction is indicated, the choice of surgical technique should be dependent on the patient's skeletal age and remaining growth to minimize the risk of iatrogenic injury to the physes. The knee joint experiences rapid growth during the first 5 years of life, followed by slower growth from age 5 until the onset of puberty, when a growth spurt occurs. The distal femoral growth plates expand by approximately 1.0 cm per year during this period between the age of 5 and the start of puberty. At puberty, this growth rate increases to around 1.2 cm per year. The growth pattern of the tibia closely mirrors that of the femur. According to Menelaus [26], skeletal maturity of the distal femur and proximal tibia is typically reached by the age of 16 in boys and by the age of 14 in girls.

**Figure 1:**
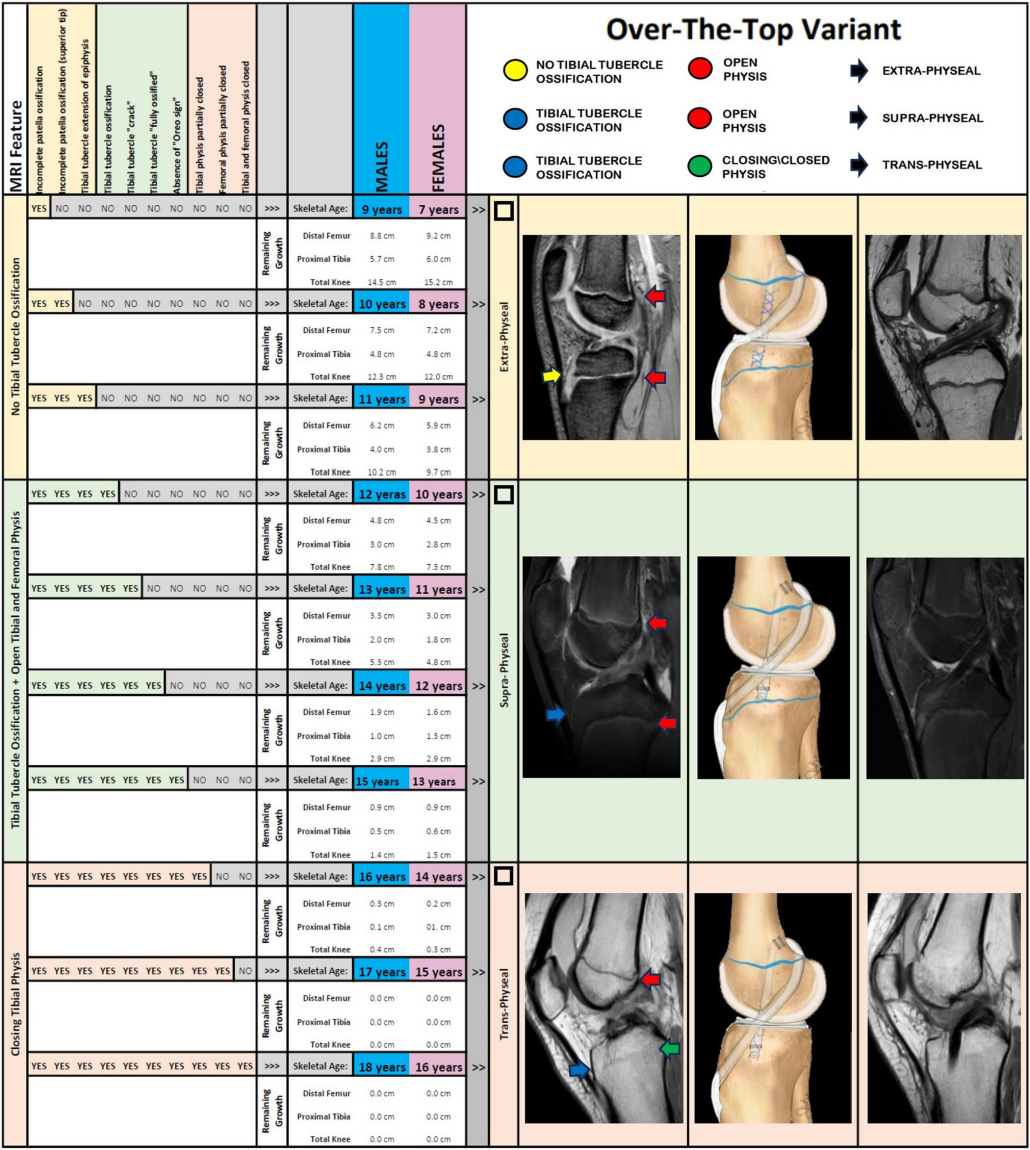
Flowchart for the surgical decision‐making for ACL rupture in skeletally immature patients, based on MRI features and skeletal age. ACL, anterior cruciate ligament; MRI, magnetic resonance imaging.

To assess bone age and guide surgical decisions, the authors recommend using the technique described by Pennock et al. [31]. By examining specific features of the knee magnetic resonance imaging (MRI), clinicians can determine the patient's bone age, with reliability comparable to the Greulich and Pyle atlas based on hand radiographs [15]. The two main features of the knee MRI used for surgical decision‐making are:

1. The presence of the tibial tubercle apophyseal centre, which is identified on sagittal T1‐weighted MRI slices by the appearance of a discrete ossification nucleus below the tibial physis, indicating that the tibial tubercle is beginning to develop (Figure 1).
2. Status of tibial and femoral growth cartilage, which is assessed on coronal T1‐weighted MRI slices, where the progression of growth cartilage is evaluated from the outer edge to the centre of the bone. The cartilage can be categorized as ‘completely visible’, ‘partially closed’ (typically in the central portion) or ‘completely closed’. Tibial cartilage generally closes earlier than femoral cartilage, so evaluating the status of both can help refine the assessment of skeletal maturity (Figure 1).

## OTT OPTIONS BASED ON MRI SKELETAL AGE

Based on combinations of these MRI features, the authors perform the following variations of the OTT technique in three different age groups. For each technique, a standard harvest of both the semitendinosus and gracilis is performed utilizing an open tendon stripper and leaving them attached to the tibial insertion. The two tendons are then whipstitched together, making sure they have equal tension, to create one graft. In all techniques, femoral fixation is performed via the OTT approach without an osseous tunnel. The graft is passed through the notch, fixed on the femur just distal to the femoral physis with the knee at 70° of flexion and slight tibial external rotation. The lateral extra‐articular tenodesis is then completed with the remnant of the hamstring graft, routed beneath the ITB, and fixed at Gerdy's tubercle on the tibia. Non‐absorbable sutures are used to fix the graft in the prepubescent group, while metal staples are used in young and old adolescent patients.

### Prepubescents (males <12 years and females <10 years of skeletal age)

The primary MRI criteria for this group is the absence of the tibial tubercle apophyseal centre, with both tibial and femoral physes completely visible. These patients are still 1 or more years away from puberty onset, with remaining growth at the knee exceeding 7.5 cm, especially on the distal femur (5 cm). At least 5 years remain until knee skeletal maturity.

### Young adolescents (males 12–15 years and females 10–13 years of skeletal age)

The key MRI criterion for this group is the presence of the tibial tubercle apophyseal centre, with both tibial and femoral physes fully visible. These patients are either close to or in the first phase of the pubertal growth spurt. The remaining growth at the knee is between 1 and 7.5 cm, with a significant amount remaining on the distal femoral side (0.5–3 cm). About 1–4 years remain until the knee is skeletal mature.

### Old adolescents (males 16–18 years and females 14–16 years of skeletal age)

The defining MRI feature for this group is the presence of a partially or completely closed tibial physis, while the femoral physis remains fully visible. These patients have completed or are nearing the end of puberty, with less than 1 cm of remaining growth at the knee. They are close to knee skeletal maturity.

#### Prepubescent patients: The ‘extra‐physeal’ technique (males <12 years and females <10 years of skeletal age)

The primary MRI criterion for this group is the absence of the tibial tubercle apophyseal centre, with both tibial and femoral physes completely visible. These patients are still 1 or more years away from puberty onset, with remaining growth at the knee exceeding 7.5 cm, especially on the distal femur (5 cm). At least 5 years remain until knee skeletal maturity.

**Figure 2:**
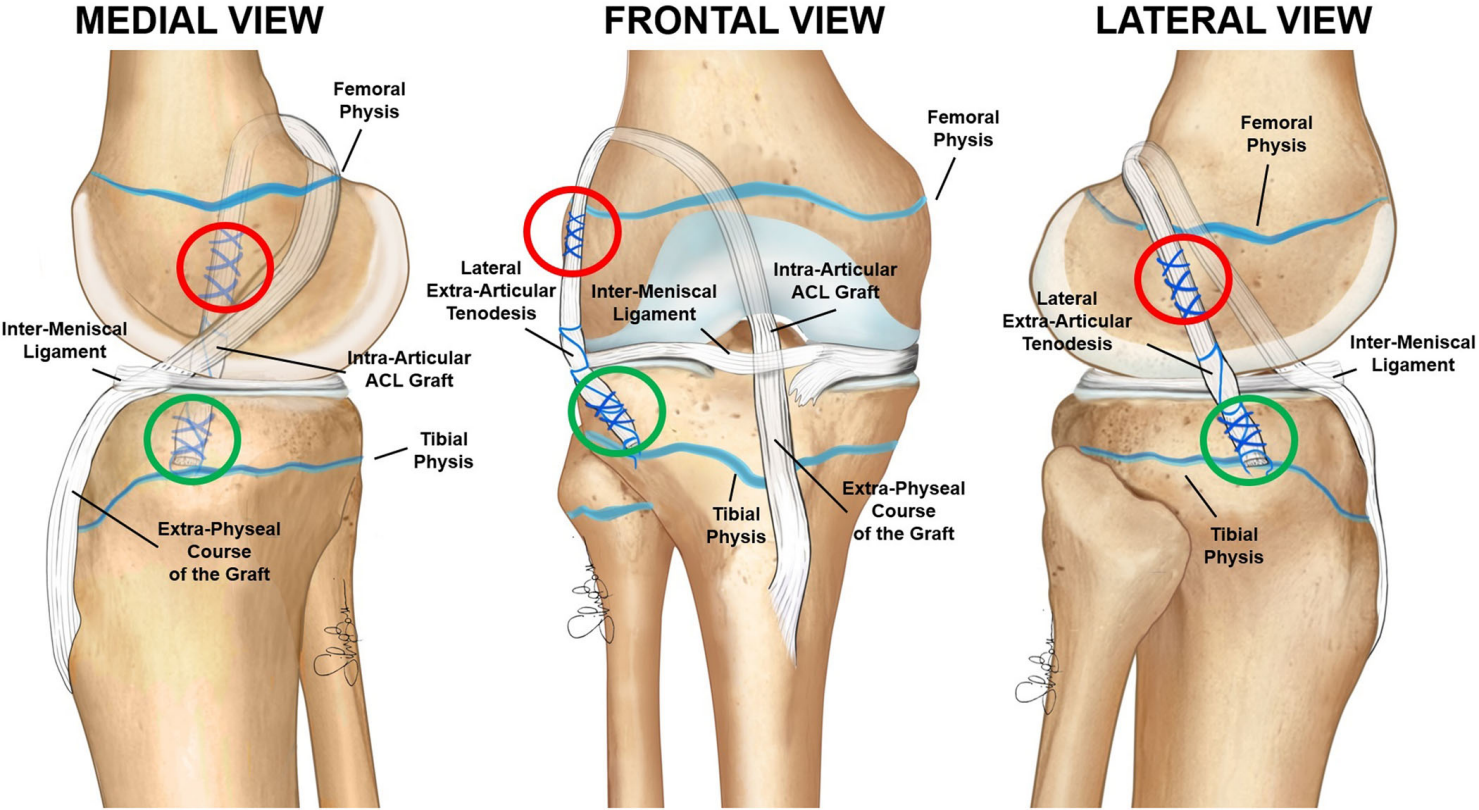
Schematic drawings of the extra‐physeal over‐the‐top ACL reconstruction technique. ACL, anterior cruciate ligament.

**Figure 3:**
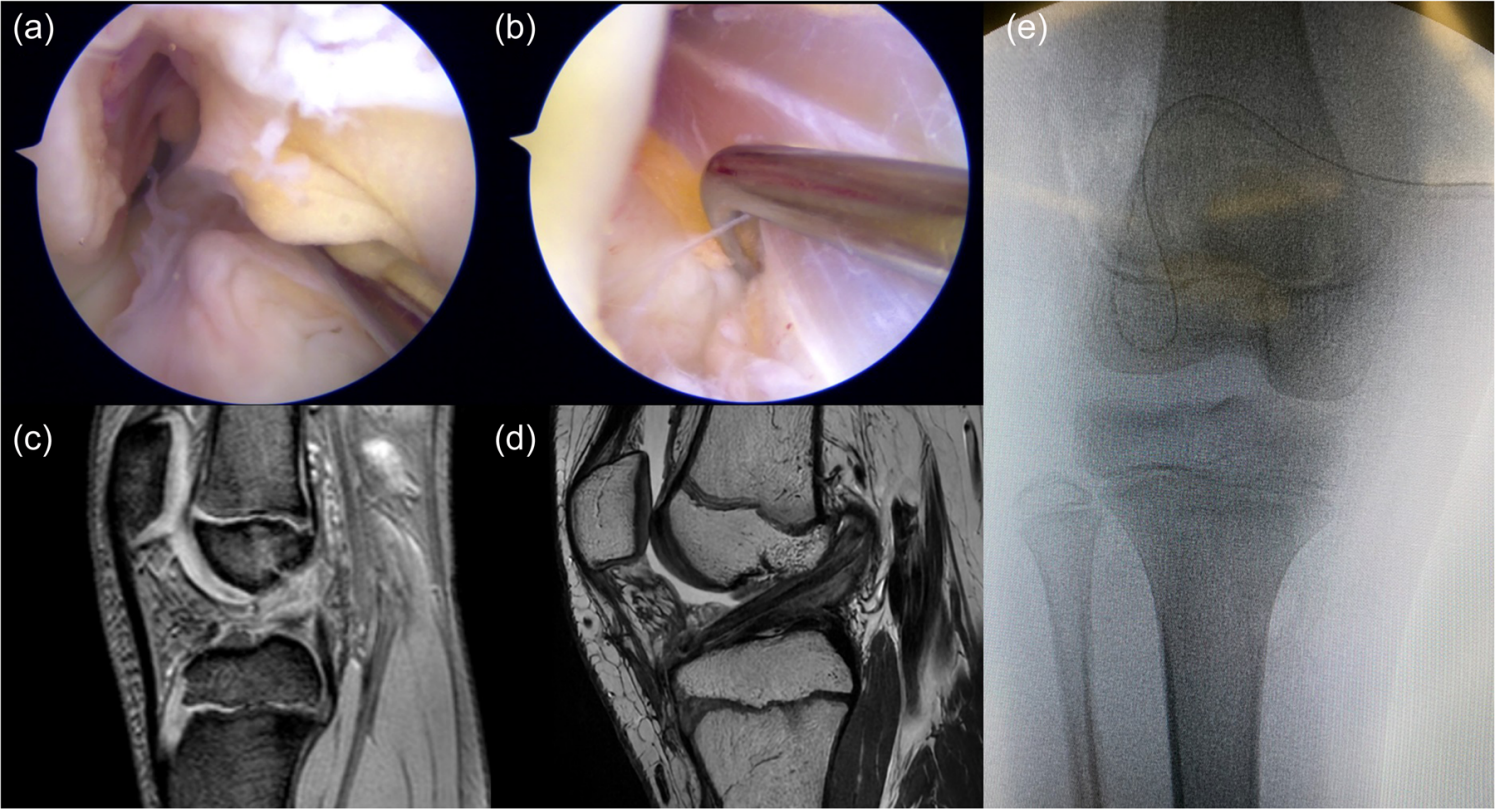
A male football player with chronological age of 11.2 years and skeletal age of 11 years was treated with extra‐physeal over‐the‐top ACL reconstruction. Arthroscopic vision with the empty notch due to ACL injury (a) and after reconstruction with hamstring graft under the intermeniscal ligament (b). Sagittal pre‐operative MRI shows the absence of the ACL (c) and the 4‐month MRI shows the graft with a good ipointense signal and the extra‐physeal oblique course, with no tibial tunnel (d). Antero‐posterior radiograph shows the absence of tunnels or hardware (e). ACL, anterior cruciate ligament; MRI, magnetic resonance imaging.

**Figure 4:**
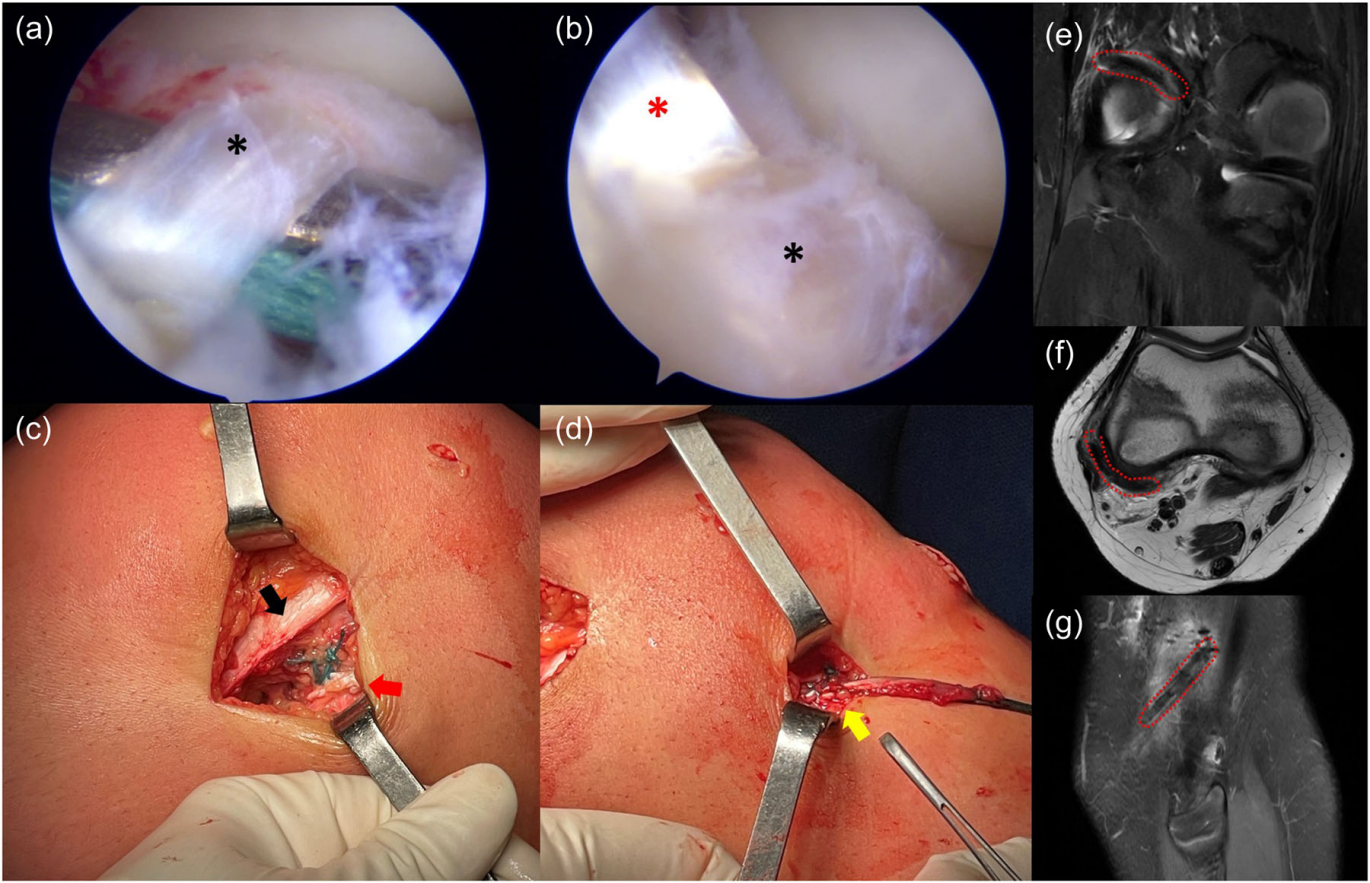
The Kelly clamp and a suture loop are passed below the intermeniscal ligament (black asterisk) (a). The hamstring graft (red asterisk) is passed below the intermeniscal ligament (black asterisk) as well (b). The graft (red arrow) is sutured extra‐articularly to the periosteum of the lateral distal femur with nonabsorbable sutures (black arrow) (c). The lateral tenodesis is sutured at the level of Gerdy's tubercle with nonabsorbable sutures (yellow arrow) (d). Post‐operative MRI shows the extra‐articular course of the graft above the lateral femoral condyle in the coronal (e) and axial (f) views, while the course of the lateral tenodesis can be seen in the sagittal view (g). MRI, magnetic resonance imaging.

For these reasons, drilling a tibial tunnel through the cartilaginous portion of the epiphysis is avoided, as is placing metal hardware near the femoral physis, due to the potential for proximal migration and stretching of the tenodesis with growth (Figure 2). Instead, the OTT and lateral tenodesis are performed in an ‘extra‐physeal’ manner, without bone tunnels (Figure 3). This approach is similar to the Micheli technique [27], but executed in a reverse fashion, with the hamstring graft passed under the intermeniscal ligament before being secured first to the lateral femur and then to the tibia at Gerdy's tubercle utilizing periosteal sutures (Figure 4).

#### Young adolescents: The ‘supra‐physeal’ technique (males 12–15 years and females 10–13 years of skeletal age)

The key MRI criterion for this group is the presence of the tibial tubercle apophyseal centre, with both tibial and femoral physes fully visible. These patients are either close to or in the first phase of the pubertal growth spurt. The remaining growth at the knee is between 1 and 7.5 cm, with a significant amount remaining on the distal femoral side (0.5–3 cm). About 1–4 years remain until the knee is skeletal mature.

**Figure 5:**
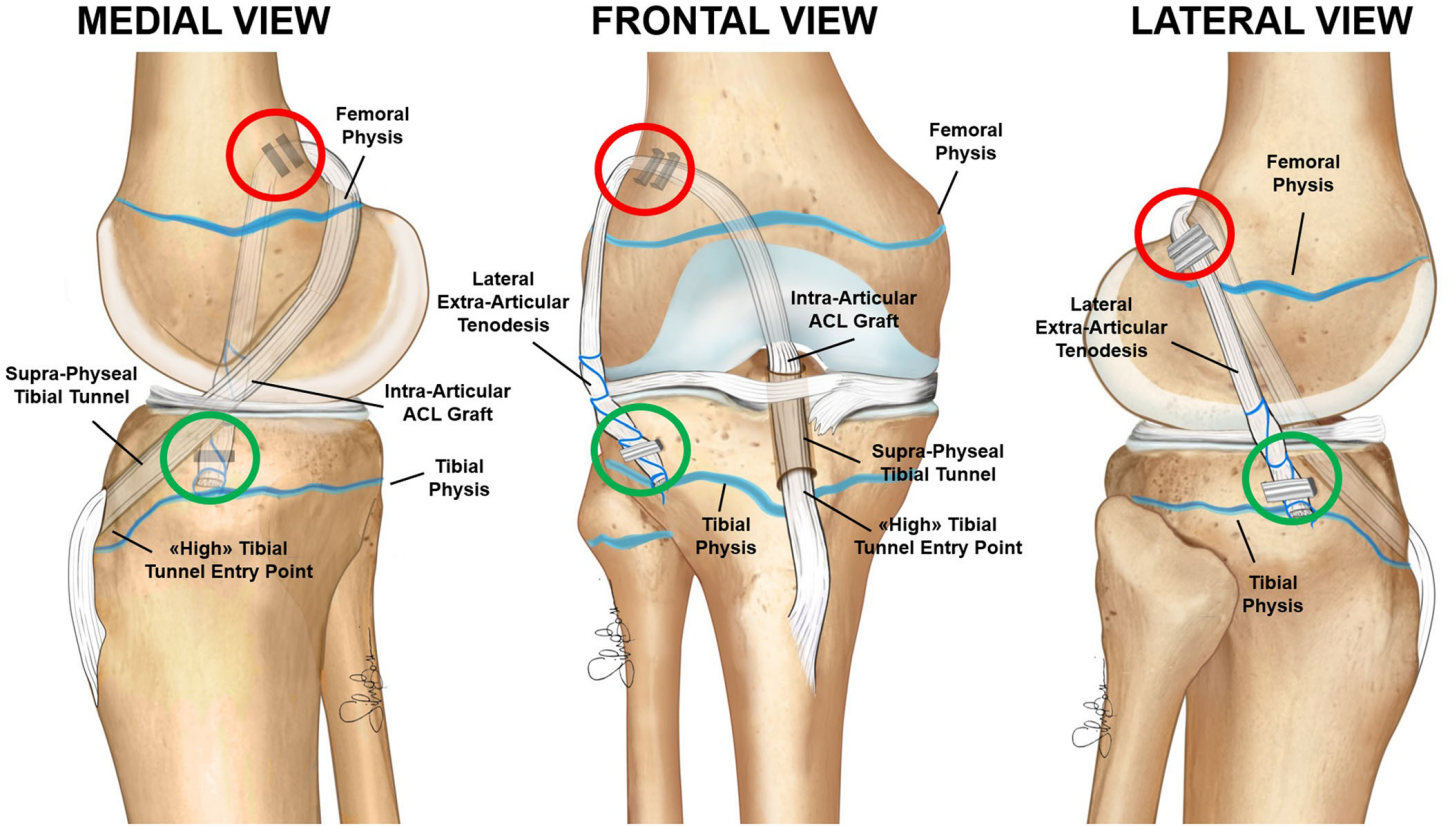
Schematic drawings of the supra‐physeal over‐the‐top ACL reconstruction technique. ACL, ACL, anterior cruciate ligament.

**Figure 6:**
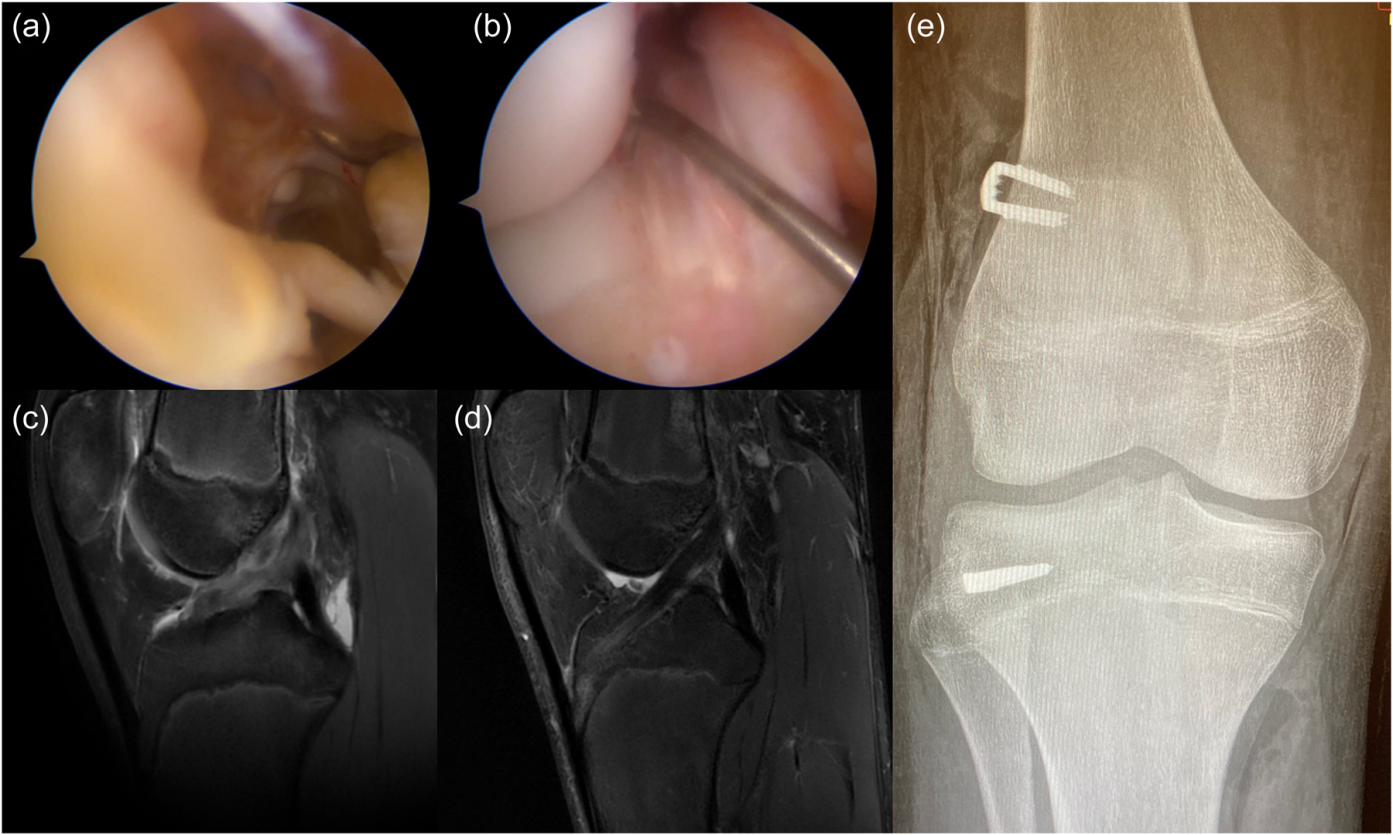
A male football player with chronological age of 14.2 years and skeletal age of 14 years was treated with supra‐physeal over‐the‐top ACL reconstruction. Arthroscopic vision with the empty notch due to ACL injury (a) and after reconstruction with hamstring graft (b). Sagittal pre‐operative MRI shows the absence of the ACL (c) and 6‐month MRI shows the graft with a good ipointense signal and the course above the growth plate and within the proximal epiphysis (d). Antero‐posterior radiograph shows no conflict of the staples with the growth plates (e). ACL, anterior cruciate ligament.

**Figure 7:**
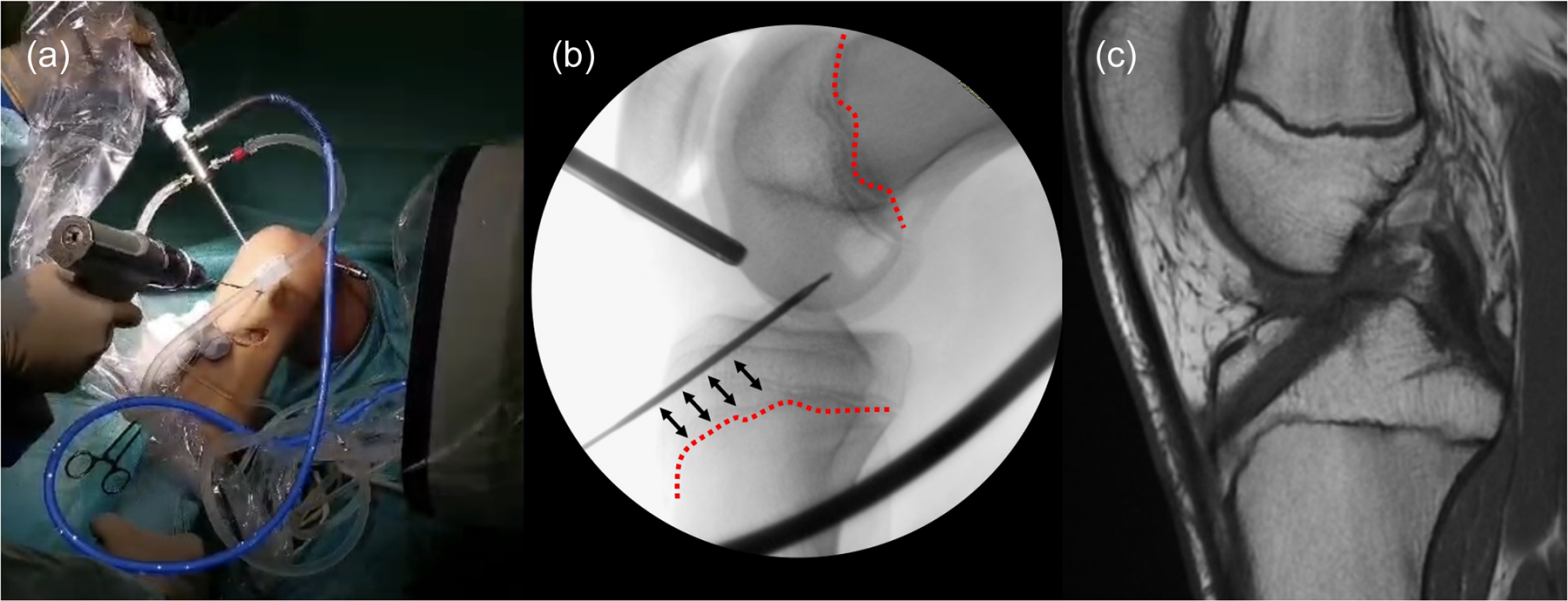
Setting for Supra‐physeal tunnel preparation, with the knee at 90° of flexion and the C‐arm perpendicular to the lower limb (a). Intra‐operative lateral x‐ray shows the placement of the k‐wire above the tibial growth plate (red dotted line) and within the proximal tibia epiphysis (b). Post‐operative MRI shows no conflict of the tibial tunnel with the tibial growth plate (c). MRI, magnetic resonance imaging.

Given the moderate amount of growth expected, the OTT and lateral tenodesis are performed in a ‘supra‐physeal’ manner (Figure 5). This involves creating a tibial tunnel proximally to the tibial physis (within the proximal tibial epiphysis) and securing the graft with staples above both the femoral and tibial physes (Figure 6). As a result, the entry point of the tibial tunnel is positioned higher than usual and requires an additional skin incision, distinct from the one used for graft harvesting. To accurately identify the correct entry point, true lateral intraoperative fluoroscopic views, with superimposed femoral condyles, are essential. This allows the surgeon to consider the three‐dimensional shape of the proximal physis and the distal expansion of the tibial tubercle ossification (Figure 7).

#### Older adolescents: The ‘trans‐physeal’ technique (males 16–18 years and females 14–16 years of skeletal age)

The defining MRI feature for this group is the presence of a partially or completely closed tibial physis, while the femoral physis remains fully visible. These patients have completed or are nearing the end of puberty, with less than 1 cm of remaining growth at the knee. They are close to knee skeletal maturity.

**Figure 8:**
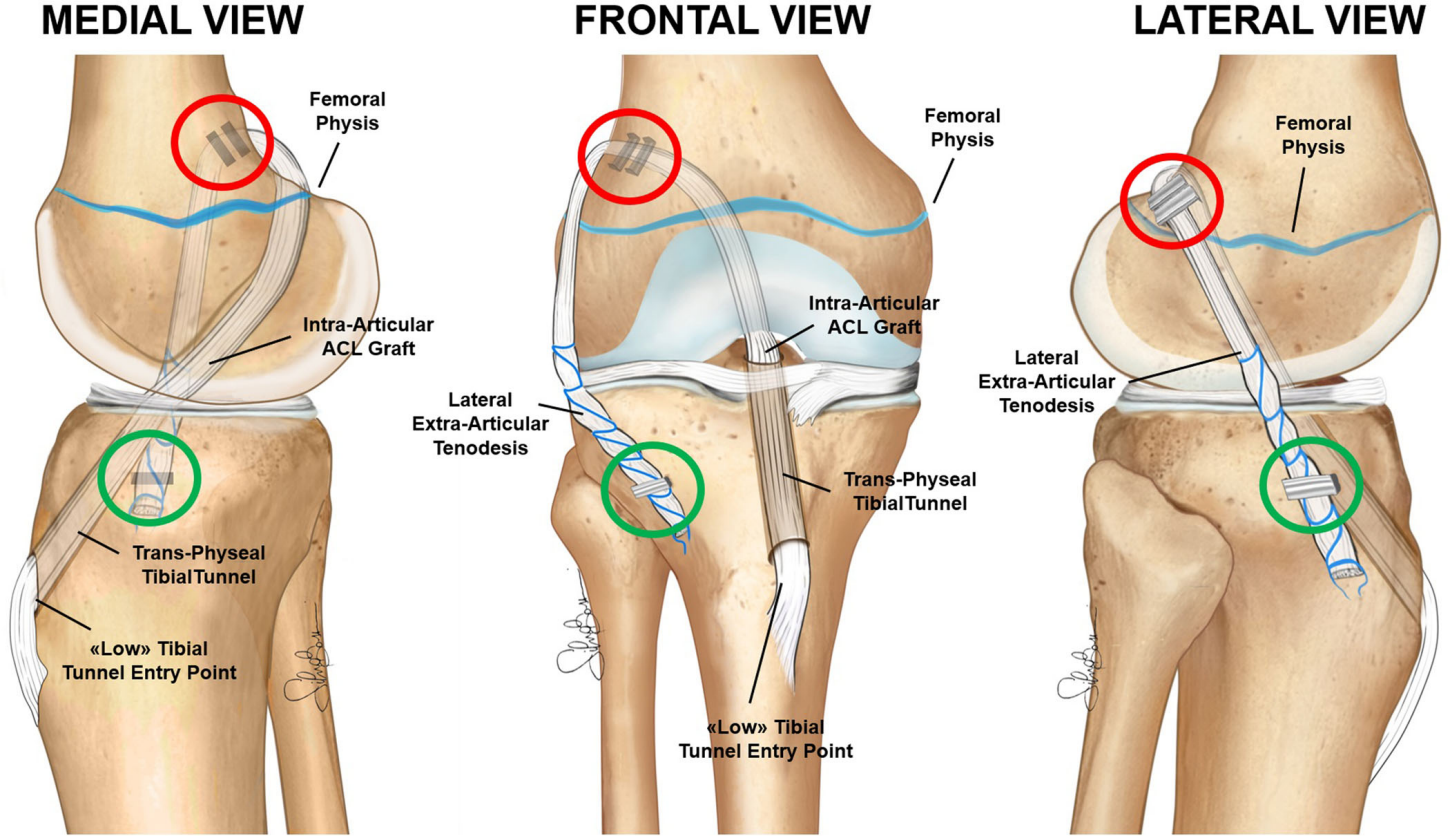
Schematic drawings of the trans‐physeal over‐the‐top ACL reconstruction technique. ACL, anterior cruciate ligament.

**Figure 9:**
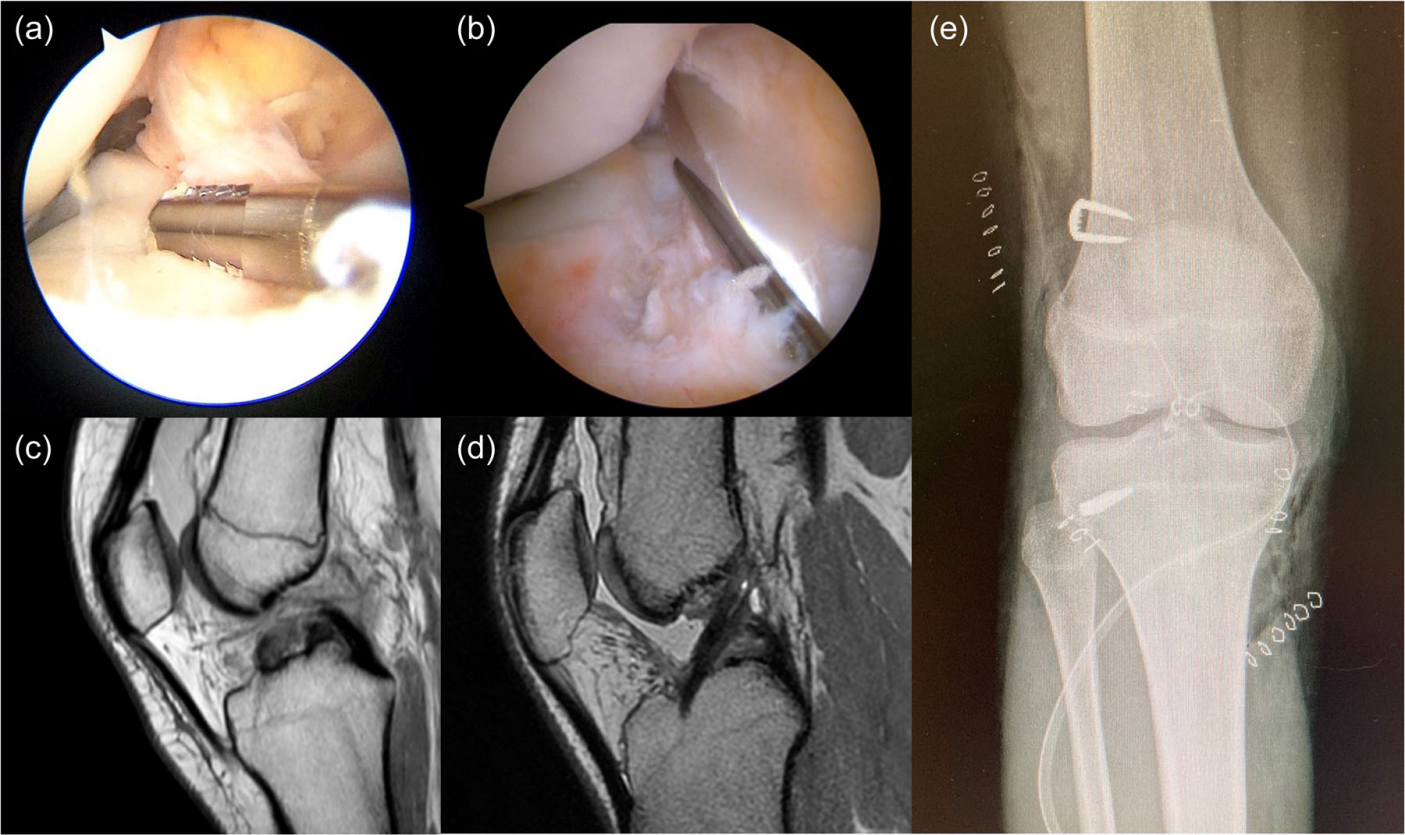
Male basketball player with chronological age of 15.8 years and skeletal age of 16 years treated with trans‐physeal over‐the‐top ACL reconstruction. Arthroscopic vision with the empty notch due to ACL injury (a) and after reconstruction with hamstring graft (b). Sagittal pre‐operative MRI shows the absence of the ACL (c) and 12‐month MRI shows the graft with a good ipointense signal (d). Antero‐posterior radiograph shows no conflict of the staples with the growth plates (e). ACL, anterior cruciate ligament.

The ‘Trans‐physeal’ technique (Figure 8) is recommended for older adolescent patients, typically males aged 16–18 years and females aged 14–16 years, when the main MRI feature is the initial closure of the tibial physis. In this group, there is typically less than 1 cm of remaining knee growth. Given the minimal remaining growth, the OTT and lateral tenodesis are performed in a ‘trans‐physeal’ manner, exactly as described in the original technique utilized in adults [27], with the tibial tunnel drilled through the closed or closing tibial physis (Figure 9).

##### Anatomical, biomechanical and biological rationale

The OTT technique offers several key advantages for the treatment of skeletally immature patients (Table 1). The most significant advantage is the ability to perform femoral fixation without the need for an osseous tunnel, which helps to avoid, or at least reduce, the risk of growth abnormalities on the femoral side. ACL reconstruction using the OTT technique has been extensively studied in cadaveric models, demonstrating its effectiveness in reducing joint laxity and maintaining satisfactory knee kinematics [22, 25, 36, 37, 40]. These findings from laboratory models have been further supported by in vivo studies using navigation systems [36] and clinical research on adult patients with both short‐ and long‐term follow‐ups [14, 23, 41, 45, 46].

**Table 1:** Ten advantages of the over‐the‐top approach.

1.	No harm to the distal femur physis
2.	Reliable and Isometric graft placement
3.	Possibility to combine lateral tenodesis with a single graft
4.	Good cosmesis and low donor site morbidity
5.	Improved graft maturation when preserving the hamstring attachment
6.	Small diameter of tibial tunnel
7.	Complete tunnel filling with graft
8.	Immediate secure cortical fixation
9.	Solid biomechanic rationale
10.	Good outcomes in adult and skeletally immature populations

*Note*: List of the 10 main advantages of the over‐the‐top techniques for the treatment of skeletally immature patients.

From a biological perspective, performing combined ACL reconstruction and lateral tenodesis with hamstring tendons, as in Marcacci's technique, offers advantages by preserving the distal tibial insertion [45]. When the gracilis and semitendinosus are harvested using an open tendon stripper without detaching the distal attachment, the neurovascular supply to the tibial insertion can be maintained. Animal models and clinical studies in humans have shown that this approach may bypass the typical necrosis and revascularization phases that the graft undergoes during the ‘ligamentization’ process, which normally occurs when tendons are detached, interrupting their neurovascular supply [39, 45]. In fact, preserving the hamstring insertion during ACL reconstruction has been associated with improved MRI findings at 4‐ and 18‐month follow‐ups, including better graft signal, less tunnel enlargement, and reduced effusion. These benefits suggest potential advantages in terms of both graft growth and mechanical properties [11].

## CLINICAL OUTCOMES AND COMPLICATIONS

### General population

The OTT and lateral tenodesis technique for ACL reconstruction has been extensively studied in the general population. Several prospective series with follow‐ups ranging from 2 to 10 years [6, 9, 14, 44] have reported excellent stability outcomes in 93.3% of patients, with a mean side‐to‐side difference of 2.1 mm as measured by the KT‐2000 arthrometer [45]. Additionally, 90% of patients returned to their pre‐injury activity level. A retrospective study of 267 consecutive cases showed a 10‐year revision rate of 3.7%. The Knee injury and Osteoarthritis Outcome Score was substantially comparable to the 10‐year results of the Multicenter Orthopaedic Outcome Network ACL registry [14].

Long‐term outcomes demonstrated that good results were maintained even after 24 years post‐surgery, with an average Lysholm score of 85.7 ± 14.6, a low rate of osteoarthritis, and 86% of patients exhibiting a normal or nearly normal knee. A large‐scale safety assessment of 2559 consecutive patients reported a 90‐day re‐admission rate of 2.3%, primarily due to nonspecific knee swelling (0.78%), superficial infections (0.63%), deep infections (0.55%) and joint stiffness (0.23%) [12].

### Skeletally immature patients

While no clinical studies have specifically evaluated the ‘extra‐physeal’ OTT technique, earlier studies by Paul Brief and Parker et al. examined similar ACL reconstructions in skeletally immature patients. Both studies reported good clinical outcomes, with high rates of return to sport and no growth disturbances [4, 29]. In early studies, the technique involved sliding the hamstring graft under the inter‐meniscal ligament or the anterior horn of the medial meniscus, and extra‐articular fixation on the lateral femur. However, these procedures were performed via medial arthrotomy, and lateral tenodesis was not routinely included in Parker's study. The ‘extra‐physeal’ technique described here, however, is performed arthroscopically with a lateral tenodesis using the same hamstring graft.

Similar principles were adopted by Kocher [20], who described an extra‐physeal technique using a long strip of ITB for lateral tenodesis. In their study of 237 children with an average age of 11.2 ± 1.7 years, they reported a failure rate of 6%, a mean Lysholm score of 93.4 ± 9.9 and a 96.5% return‐to‐sport rate [19]. However, nearly 50% of patients reported asymmetry in the lateral thigh, with a muscle ‘bump’ or ‘fullness’ that could be avoided by using hamstrings instead of ITB for lateral tenodesis. Another study of the Micheli technique reported a higher failure rate (14%) but confirmed the safety of the technique with no growth disturbances after 3 years of follow‐up [43].

Anecdotal reports of growth abnormalities associated with the OTT approach were primarily confined to cases involving transphyseal tibial tunnels or the use of cortical femoral hardware. These abnormalities typically manifested as symmetrical overgrowth (Table 2) and were likely caused by bone hypervascularization and subperiosteal damage during fixation [7, 47]. This underscores the potential benefits of minimizing bone aggression in skeletally immature patients with significant growth potential. This evidence supports the use of the ‘extra‐physeal’ OTT and lateral tenodesis technique as an effective and safe option for treating very young and prepubescent patients.

**Table 2:** Growth disturbances with over‐the‐top (OTT) approach.

Patient details					Surgical technique							Growth disturbance				
Authors	Year	Chronological age	Sex	Injury	Concomitant surgery	Graft	Tibial tunnel	Tibial fixation	Femoral tunnel	Femoral fixation	Lateral tenodesis	Femoral	Tibia	Total	Aligment	Type
Zimmerman et al.	2015	11.0 years	M	Lacrosse (non‐contact)	Lateral meniscus repair	Posterior tibialis allograft	Transphyseal	Screw post	No (OTT)	Screw post	No	+1.8 cm	+1.0 cm	+2.8 cm	0°	Overgrowth
Rozburch et al.	2013	12.0 years	M	Skiing	No	Achilles allograft	Transphyseal	Cortical button	No (OTT)	Staples	No	Mainly tibia		+4.5 cm	15° varus	Overgrowth + varus + recurvatum
Chotel et al.	2010	7.0 years	M	‐	Medial meniscus repair	ITB autograft	Transphyseal (6 mm)	Staple	No (OTT)	No	Yes (under LCL)	50% femur, 50% tibia		+1.5 cm	0°	Overgrowth
		10.5 years	M	‐	No	ITB autograft	Transphyseal (6 mm)	Staple	No (OTT)	No	Yes (under LCL)	+0.0 cm	+1.0 cm	+1.0 cm	6° valgus	Overgrowth + valgus
Andrews et al.	1994	15.0 years	M	Football (contact)	Lateral meniscus repair	ITB allograft	Transphyseal (6–7 mm)	Staple	No (OTT)	Staples	No	−1.0 cm	+0.0 cm	−1.0 cm	0°	Shortening
		15.0 years	M	Soccer (contact)	Lateral meniscus repair	ITB allograft	Transphyseal (6–7 mm)	Staple	No (OTT)	Staples	No	+1.0 cm	+0.0 cm	+1.0 cm	0°	Overgrowth
		9.0 years	M	Football (non‐contact)	Medial meniscus repair	ITB allograft	Transphyseal (6–7 mm)	Staple	No (OTT)	Staples	No	−1.2 cm	+0.0 cm	−1.2 cm	0°	Shortening
Roberti di Sarsina et al.	2019	8.8 years	F	Artistic Gymnast	Lateral meniscus repair	Hamstrings	Supra‐physeal (6 mm)	No	No (OTT)	Staples	Yes (above LCL)	Mainly femur		+0.6 cm	4° varus	Overgrowth + varus
		11 years	M	Soccer (non‐contact)	Medial meniscus removal	Hamstrings	Supra‐physeal (6 mm)	No	No (OTT)	Staples	Yes (above LCL)	+0.0 cm	+0.0 cm	+0.0 cm	3° varus	Varus
		13.7 years	M	Soccer (non‐contact)	Medial meniscus repair	Hamstrings	Supra‐physeal (6 mm)	No	No (OTT)	Staples	Yes (above LCL)	Mainly femur		+1.0 cm	0°	Overgrowth

*Note*: Growth disturbances of ACL reconstruction using the OTT approach.

Abbreviations: ACL, anterior cruciate ligament; ITB, iliotibial band; LCL, lateral collateral ligament.

### The ‘supra‐physeal’ OTT technique

Mid‐term results of the ‘supra‐physeal’ OTT technique have been reported in a series of 20 patients aged 14 years or younger [35], which includes both prepubescent and young adolescent patients. At an average follow‐up of 4.5 years, no failures requiring revision were reported. The overall patient‐reported outcome measures and sport participation were generally rated as good to excellent. A total of three (15%) minor growth disturbances (3–4° of varus deviation) were reported, with one (5%) of these patients having a sub‐optimal result (International Knee Documentation Committee B) as well. Two of these three patients were prepubescent at the time of surgery.

A larger study of 132 adolescent patients found a 26% rate of staple removal due to local discomfort in patients under 14 years of age, compared to only 4% in those between 14 and 16 years old [13]. These results suggest that the modified OTT technique without bone tunnels and metal staples may be more appropriate for prepubescent patients to avoid complications.

### Outcomes in older adolescents

An internal registry study of 168 male patients aged 16–18 and female patients aged 14–16 showed a failure rate of 9% within the first 2 years of follow‐up, increasing to 13% after an average follow‐up of 6.9 ± 3.4 years [10]. Additionally, 15% of patients in this cohort underwent contralateral ACL reconstruction during the follow‐up period. These findings align with the generally higher failure rates observed in this high‐risk age group [1, 2, 8, 33].

## CONCLUSIONS

Different modifications of the OTT and lateral tenodesis techniques using hamstring tendons offer promising solutions for treating ACL injuries in skeletally immature patients, spanning from the prepubescent phase to late adolescence. It is crucial to identify the skeletal age, and thus the remaining bone growth, to determine the optimal surgical approach. The biomechanical rationale and favourable clinical outcomes in various populations support this approach as an appealing, effective, and safe option for managing these complex cases.

## AUTHOR CONTRIBUTIONS

All authors contributed to the study conception and design. Material preparation and analysis were performed by Alberto Grassi, Matthew Tao and Stefano Zaffagnini. The first draft of the manuscript was written by Alberto Grassi and all authors commented on previous versions of the manuscript. All authors read and approved the final manuscript.

## CONFLICT OF INTEREST STATEMENT

The authors declare no conflicts of interest.

## ETHICS STATEMENT

This article does not contain any studies with human participants or animals performed by any of the authors.

## Data Availability

No data are available from this manuscript since it is a current concepts review and does not contain any data collected for this manuscript.
